# CXCL13 shapes tumor immune microenvironment in ovarian cancer with homologous recombination deficiency

**DOI:** 10.1016/j.gendis.2023.101200

**Published:** 2023-12-19

**Authors:** Yue Ding, Zheng Ye, Bo Ding, Songwei Feng, Furong Du, Xuejiao Ma, Xiaoxuan Wang, Yang Shen

**Affiliations:** a Department of Obstetrics and Gynaecology, Zhongda Hospital, School of Medicine, Southeast University, Nanjing, Jiangsu 210008, China; b State Key Laboratory of Bioelectronics, School of Biological Science and Medical Engineering, Southeast University, Nanjing, Jiangsu 210096, China; c State Key Laboratory of Translational Medicine and Innovative Drug Development, Jiangsu Simcere Diagnostics Co., Ltd., Nanjing, Jiangsu 210042, China

Recently, immune checkpoint inhibitors (ICIs) and poly (ADP-ribose) polymerase inhibitors (PARPi) have played a pivotal role in prolonging the recurrence-free survival of patients with ovarian cancer (OC).^1^^,^^2^ Although PARPi have revolutionized the treatment of OC, the absence of reliable predictive biomarkers limits the broad application of ICIs for patients with homologous recombination (HR) deficiency (HRD).^3^ CXC-chemokine ligand 13 (CXCL13) is a cytokine constitutively secreted in the stromal cells of the B-cell region of secondary lymphoid tissue.^4^ It exclusively binds to the chemokine receptor CXCR5, which is abundantly expressed in subsets of mature circulating B lymphocytes, follicular helper T cells, and skin-derived dendritic cells, and governs the migration of these cells into secondary lymphoid organs in response to the CXCL13 gradient.^5^ In this study, we aimed to elucidate the role of CXCL13 in shaping the tumor immune microenvironment (TIME) in HR-deficient OC and to explore its relationship with the cGAS-STING signaling pathway.

Due to the absence of matched para-cancerous tissues in The Cancer Genome Atlas (TCGA) cohort, we utilized the GTEx database of normal ovarian samples to analyze CXCL13 expression. The expression of CXCL13 was significantly higher in cancer compared with normal tissues (Fig. 1A). In addition, we obtained a similar conclusion at the protein expression level (Fig. S1A). The effect of CXCL13 on the prognosis reveals that patients with high CXCL13 expression had superior overall survival (Fig. 1B; Log-rank *P* = 0.002), and high CXCL13 expression was an independent prognostic factor (Fig. S1B). The analysis using the Gene Expression Omnibus dataset yielded similar results (Fig. S1C). Moreover, we confirmed that HRD status was closely related to CXCL13 in 20 FFPE samples (HRD-H: 15; HRD-L: 5) (Fig. S2A). The clinical characteristics of all tissue samples are summarized in Table S1. We collected more than 100 genes from the cGAS-STING pathway and 45 genes from the HR repair (HRR) pathway to analyze their relationship (Fig. S2B, 3). BRCA1 plays a pivotal role in linking the cGAS-STING and HRR pathways, as we have discovered (Fig. S2C).

**Figure 1:**
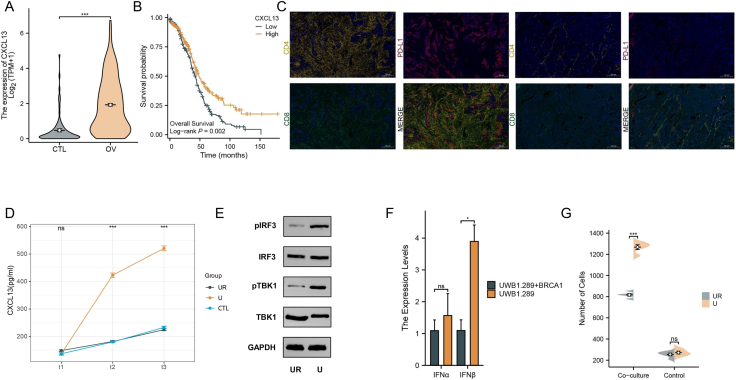
CXCL13 plays a critical role in shaping the anti-tumor microenvironment by recruiting CD8^+^ T cells and could serve as a predictive biomarker for response to immune checkpoint inhibitors in HR-deficient ovarian cancer (OC). **(A)** Differential expression of CXCL13 in OC. **(B)** The curve for overall survival is shown for high and low CXCL13 expression. **(C)** CXCL13 co-localizes with CD4^+^ and CD8^+^ T cells. Left: Representative immunofluorescence staining of HGSOC with HRD. Right: Representative immunofluorescence staining of HGSOC with non-HRD. **(D)** Differential expression of CXCL13 at the protein level (UR: UWB1.289+BRCA1 & CD4^+^ T; U: UWB1.289 & CD4^+^ T; CTL: only CD4^+^ T). **(E)** The expression of cGAS-STING pathway proteins (confirmed by Western blot). **(F)** Enzyme-linked immunosorbent assay (ELISA) results. **(G)** Effects of CXCL13 expression on migration of the CD8^+^ T cells. Migrated cell numbers in three different fields (each field = 200 × 200 μm) were counted in the experiment and the values were averaged. Three independent experiments were performed. The bars represent mean ± standard error of the mean. ^∗^*P* < 0.05, ^∗∗^*P* < 0.01, ^∗∗∗^*P* < 0.001. ns, not statistically significant; HR, homologous recombination; HRD, homologous recombination deficiency.

As shown in Figure S4A, there was a significant positive correlation between CXCL13 expression and immune scores (*r* > 0.6), suggesting that CXCL13 may play a critical role in the regulation of TIME. Using the TIMER and xCell algorithms, we found that CXCL13 was strongly correlated with neutrophils and dendritic cells, and weakly correlated with B cells, T cells, and macrophages (Fig. S4B). Additionally, using other algorithms, we observed a positive correlation between CXCL13 expression and most immune cells, particularly CD4^+^ and CD8^+^ T cells (Fig. S5). Then we found that CXCL13 was highly correlated with PDCD1, CD8A, and CTLA4 in depleted T cells at the single-cell level, indicating that CXCL13 may serve as a stable biomarker for immunotherapy (Fig. S6A–D).

Multiplex immunohistochemistry results confirmed that the distribution of CXCL13 was close to CD4^+^ T and CD8^+^ T cells, together with PD-L1 expression (Fig. 1C). The conglomerate of cell density increased with high CXCL13 levels, indicating that CXCL13 may contribute to CD8^+^ T cell agglomeration in the TIME. We observed colocalization between CXCL13 and CD4^+^ T cells, indicating that CXCL13 may be sourced from CD4^+^ T cells. Consistent with expectations, we found that CXCL13 levels were significantly raised in the supernatants collected after co-culture (Fig. 1D). The phosphorylation levels of TBK1 and IRF3 were significantly increased in UWB1.289 (Fig. 1E; Fig. S5E). Additionally, the small molecule inhibitor of the cGAS-STING pathway can suppress the secretion of interferon beta (IFNβ) in UWB1.289 cells (Fig. S7A). These results suggest the activation status of the signaling pathways. The activation of the cGAS-STING signaling pathway could result in IFN1 release, which caused subsequent changes in the TIME. To determine the subtypes of type I interferon that acts directly on CD4^+^ T cells, IFNα/β in the coculture supernatant containing an equal number of cells was analyzed by ELISA. We found that IFNβ levels were elevated in the supernatant, while IFNα did not change obviously (Fig. 1F). To validate the result, CD4^+^ T cells were treated with IFNβ for 24 h and CXCL13 was significantly increased (Fig. S7B). After adding an IFNβ inhibitor to the co-culture system, there was a significant decrease in CXCL13 secretion (Fig. S7C). To evaluate the chemotactic ability of CXCL13 to CD8^+^ T cells, we used the Transwell device (Fig. S7D) and observed that the average number of cells per field that was able to cross the membrane was higher in the UWB1.289 and CXCL13 groups, but not in the UWB1.289+BRCA group (Fig. S7E). Statistics confirmed the correlation between CXCL13 and CD8^+^ T cells in patients with HR-deficient OC (Fig. 1G).

Our results demonstrate that CXCL13 confers anti-tumor effects via the recruitment of CD8^+^ T cells, and its expression depends on the activation of the cGAS-STING pathway. CXCL13 could also be a marker to assess the suitability of immunotherapy for HRD-positive patients who have a poor response to PARP inhibitors. This study is not without its limitations, notably the use of a single antibody in the immunohistochemistry process and the inclusion of a limited number of cases. Consequently, future research should consider the use of well-characterized antibodies and a more extensive cohort.

## Ethics declaration

The study was approved by the IEC for Clinical Research of Zhongda Hospital, Affiliated to Southeast University (approval number: 2021ZDSYLL177-P01). Written informed consent was obtained from each participant.

## Author contributions

YS conceived and designed the study. YD and ZY analyzed and interpreted the data. YD and ZY drafted the manuscript. YD, BD, and SF performed the acquisition, analysis, and interpretation of the data and statistical analysis. YS and FD revised the manuscript critically. FD, XM, and XW provided technical support. All authors approved the final draft of the manuscript.

## Conflict of interets

All authors declare no conflict of interests. The funders had no role in the design of the study, the collection, analyses, or interpretation of data, the writing of the manuscript, or the decision to publish the results.

## Funding

This work was funded by the Beijing Xisike Clinical Oncology Research Foundation (China) (No. Y-zai2022/ms-0126).

## Data availability

These data were derived from the following resources available in the public domain (list in *Materials and Methods*). Further details and other data that support the findings of this study are available from the corresponding author upon request.

## Appendix A Supplementary data

Multimedia component 2 Multimedia component 2

Multimedia component 3 **Figure S1** CXCL13 is a good prognostic biomarker for ovarian cancer (OC). **(A)** Expression of CXCL13 in pan-cancers (paired samples). **(B)** A forest plot on the association between CXCL13 expression and overall survival. **(C)** Kaplan-Meier survival curve (disease-specific survival/DSS and progression-free interval/PFI) for different expression statuses of CXCL13. ^∗^*P* < 0.05, ^∗∗^*P* < 0.01, ^∗∗∗^*P* < 0.001. ns, not statistically significant. **Figure S2** Multiomics analysis of the relationship between CXCL13 and HRD. **(A)** The expression of CXCL13 protein in clinical specimens with different HRD statuses (200×). **(B)** Correlation between CXCL13 expression and gene expression levels of 45 HRRs. **(C)** Protein-protein interaction (PPI) network of related genes co-expressed with CXCL13 obtained by the STRING tool. ^∗^*P* < 0.05, ^∗∗^*P* < 0.01, ^∗∗∗^*P* < 0.001. ns, not statistically significant; HRD, homologous recombination deficiency; HRR, homologous recombination repair. **Figure S3** Co-expressed genes and mutational landscape. **(A–C)** Correlation analysis of CXCL13 expression with gene expression of the cGAS-STING pathway. **(D)** The top 15 most highly mutated genes between CXCL13-H and CXCL13-L groups. **Figure S4** Correlation of CXCL13 expression with tumor immune microenvironment was assessed by ESTIMATE and TIMER. **(A)** Significantly higher immune scores in the CXCL13-H group. **(B)** The association of six immune cell types with CXCL13. **Figure S5** The expression of CXCL13 and its correlation with immunity in ovarian cancer cell lines. **(A–D)** Various algorithms were used to assess the tumor immune microenvironment (TIME) (A: xCell algorithm; B: CIBERSORT algorithm; C: QUANTISEQ algorithm; D: MCPcounter algorithm). **(E)** Expression of CXCL13 in the co-culture system of UWB1.289 and UWB1.289+BRCA. "U" represents UWB1.289 in the co-culture system, and "UR" represents UWB1.289+BRCA1 in the co-culture system. **Figure S6** Gene expression and GSEA (apoptosis pathway) at the single-cell level. **(A)** Annotation of single cell fractionation. **(B)** Expression of 6 genes. **(C)** GSEA analysis on a single cell level. **(D)** Levels of CXCL13 expression in multiple different cell types. **Figure S7** The source and function of abnormally elevated CXCL13 in HRD-positive ovarian cancer. **(A)** Differential expression of IFNβ at the protein level. **(B)** Effects of different IFNβ concentrations on expression of CXCL13 in CD4^+^ T cells. **(C)** Differential expression of CXCL13 at protein level. **(D)** Pattern diagram of the chemotaxis experimental grouping (U: UWB1.289 & CD8^+^ T; UR: UWB1.289+BRCA1 & CD8^+^ T). **(E)** Chemotaxis assays were performed in 12-well Transwell. ^∗^*P* < 0.05, ^∗∗^*P* < 0.01, ^∗∗∗^*P* < 0.001. ns, not statistically significant; HRD, homologous recombination deficiency; IFN, interferon. Multimedia component 3
