# Supplementary material for: CXCL13 shapes tumor immune microenvironment in ovarian cancer with homologous recombination deficiency
Source: Genes Dis. 2023 Dec 19;11(5):101200. doi: 10.1016/j.gendis.2023.101200 (PMC11167236; doi:10.1016/j.gendis.2023.101200)
Supplement: Multimedia component 1 [file mmc1.docx]

| Characteristics | HRD-H | HRD-L | P value |
| --- | --- | --- | --- |
| n | 15 | 5 |  |
| Age, mean ± sd | 54.667 ± 7.8437 | 58 ± 7.3824 | 0.415^a^ |
| FIGO Stage, n (%) |  |  | 0.147^b^ |
| II | 1 (5%) | 2 (10%) |  |
| III | 9 (45%) | 3 (15%) |  |
| IV | 5 (25%) | 0 (0%) |  |
| LOH, mean ± sd | 26.333 ± 5.4598 | 9 ± 4.8477 | < 0.001 ^a^ |
| TAI, median (IQR) | 14 (10.5, 19) | 6 (4, 12) | 0.016^c^ |
| LST, mean ± sd | 24.4 ± 7.0285 | 7.6 ± 3.5777 | < 0.001 ^a^ |
| HRD score, mean ± sd | 67.133 ± 11.173 | 23.8 ± 10.663 | < 0.001 ^a^ |
| BRCA gene mutation, n (%) |  |  | 0.260 ^b^ |
| no | 9 (45%) | 5 (25%) |  |
| yes | 6 (30%) | 0 (0%) |  |

Baseline characteristics between HRD-H and HRD-L groups

a: *t* test

b: Fisher test

c: Wilcoxon

HRD, Homologous Recombination Deficient; FIGO, International Federation of Gynecology and Obstetrics; LOH, loss of heterozygosity; LST, Large scale transitions; TAI, Telomeric-allelic imbalance; BRCA: breast cancer susceptibility gene; IQR，interquartile range.
