# Supplementary material for: CXCL13 shapes tumor immune microenvironment in ovarian cancer with homologous recombination deficiency
Source: Genes Dis. 2023 Dec 19;11(5):101200. doi: 10.1016/j.gendis.2023.101200 (PMC11167236; doi:10.1016/j.gendis.2023.101200)
Supplement: Multimedia component 2 [file mmc2.docx]

**Material and Methods**

Patients and specimens

Tissue specimens from 20 HGSOC patients who underwent surgery at Department of Gynecology and Obstetrics, Zhongda Hospital, Affiliated to Southeast University between 2018 and 2021 were obtained. None of the patients had infection, autoimmune disorders, or other neoplastic diseases. Patients who received chemotherapy, radiation, or any other antitumor treatments before tumor resection were excluded. The study was approved by the International Ethics Committee for Clinical Research of Zhongda Hospital, Affiliated to Southeast University (Approval Number: 2021ZDSYLL177-P01), and all samples were obtained with ethical clearance.

HRD score

A panel-based next-generation sequencing (NGS) assay was used to detect HRD in formalin-fixed, paraffin-embedded (FFPE) samples. DNA was extracted from unstained FFPE sections with a proportion of tumor cells greater than 20% and then purification and library preparation were performed. Double-ended sequencing on the Illumina (NextSeq 550DX/NovaSeq 6000) platform at an average depth of 1000X with a panel of 45 homologous recombination repair (HRR)-related genes was conducted after the captured library was mixed, degenerated, and diluted. HRD was diagnosed if there was a *BRCA1/2* gene mutation and/or HRD score＞40.

Cell culture

The UWB1.289 cells, Jurkat cells, and UWB1.289+BRCA1 cells from FuHeng Biology were cultured at 37°C in 5% CO2 using different media: UWB1.289 and UWB1.289+BRCA1 in 50% MEGM media + 50% RPMI-1640 medium + 3% FBS± G-418, Jurkat cells in RPMI 1640 medium. Unactivated CD4^+^ or CD8^+^ T cells were isolated from PBMCs using a positive selection isolation kit (Invitrogen Life Technologies, Grand Island, NY) and cultured in complete RPMI 1640 medium supplemented with interleukin-2 (IL-2).

Immuno-infiltration analysis

Tumor Immune Estimation Resource (TIMER) was used to analyze the relationship between immune cell infiltration and regulation of gene expression ^1^. In addition, the relative infiltration ratios of eight immune cell types were obtained by EPIC algorithm ^2^.

Immunohistochemistry (IHC)

CXCL13 antibodies were purchased from Proteintech (dilution 1:500; Cat No.10927-1-AP). To avoid bias and subjective interpretation, IHC staining images were assessed by two independent observers who were blinded to the clinical data. IHC scoring was performed as previously described ^3^.

Multiplex immunohistochemistry (mIHC)

To investigate the relationship between CXCL13 and tumor-infiltrating lymphocytes (TILs), mIHC was conducted as previously described ^4^. The primary antibodies inclued CXCL13 (dilution 1:500; Proteintech), CD4 (dilution 1:200; Sigma-Aldrich), CD8 (dilution 1:200; Cell Signaling Technology), and PD-L1 (dilution 1:750; Sigma-Aldrich). We imaged and scanned all slides using a PerkinElmer Vectra3® platform.

Transwell co-culture system

The co-culture experiment was performed in 6-well Transwell plates (0.4-μm pore size, Polycarbonate, Corning). The UWB1.289 or UWB1.289 (1×10^5^ cells/ml) cells were seeded at the bottom well and the CD4^+^T cells (1×10^5^ cells/ml) were seeded in the upper inserts. The cells were co-cultured for 36 h, without medium changing.

Enzyme-linked immunosorbent assay (ELISA)

Concentrations of soluble CXCL13, interferon (IFN) α, and IFNβ proteins were determined using commercial kits (CUSABIO) according to the manufacturer’s instructions. The experimental results were analyzed by “Curve Expert” software (https://www.cusabio.com/).

Chemotaxis assay

CD8^+^T were resuspended in RPIM 1640 and placed at a concentration of 1×10^7^ cells/mL in the upper chamber of Transwell plates (Corning). OC cells and CD4^+^T cells were plated in the lower chamber. These systems were incubated for 2 h. The chemotaxis percentage was calculated as the number of migrated cells in the lower chamber divided by the total number of cells.

Small molecule inhibitors

To investigate the activation status of the cGAS-STING pathway, UWB1.289 cells were treated with 0.4 μM of Bx795 for 24 h, with DMSO treatment as the control group. Additionally, a small molecule inhibitor CCCP was used in co-culture systems at a concentration of 50 μM for 6 h, with DMSO treatment as the control group.

Statistical analysis

We compared the differences between the two groups using either an two-sample *t*-test or a nonparametric test. Significant differences for more than two groups were determined by one-way analysis of variance (ANOVA) followed by multiple comparisons with the Bonferroni post hoc test. All statistical analyses were performed using SPSS software version 22.0, with significance levels indicated by asterisks (**p*<0.05, ***p*<0.01, and ****p*<0.001).

**References**

1. Li T, Fu J, Zeng Z, et al. TIMER2.0 for analysis of tumor-infiltrating immune cells. Nucleic Acids Res. 2020;48(W1): W509-W514. doi:10.1093/nar/gkaa407

2. Racle J, de Jonge K, Baumgaertner P, Speiser DE, Gfeller D. Simultaneous enumeration of cancer and immune cell types from bulk tumor gene expression data. Elife. 2017;6. doi:10.7554/eLife.26476

3. Yang M, Lu J, Zhang G, et al. CXCL13 shapes immunoactive tumor microenvironment and enhances the efficacy of PD-1 checkpoint blockade in high-grade serous ovarian cancer. J Immunother Cancer. 2021;9(1). doi:10.1136/jitc-2020-001136

4. Lee SW, Lee HY, Kang SW, et al. Application of Immunoprofiling Using Multiplexed Immunofluorescence Staining Identifies the Prognosis of Patients with High-Grade Serous Ovarian Cancer. Int J Mol Sci. 2021;22(17). doi:10.3390/ijms22179638
