# Supplementary material for: CXCL13 shapes tumor immune microenvironment in ovarian cancer with homologous recombination deficiency
Source: Genes Dis. 2023 Dec 19;11(5):101200. doi: 10.1016/j.gendis.2023.101200 (PMC11167236; doi:10.1016/j.gendis.2023.101200)
Supplement: Multimedia component 3 — Figure S1 CXCL13 is a good prognostic biomarker for ovarian cancer (OC). (A) Expression of CXCL13 in pan-cancers (paired samples). (B) A forest plot on the association between CXCL13 expression and overall survival. (C) Kaplan-Meier survival curve (disease-specific survival/DSS and progression-free interval/PFI) for different expression statuses of CXCL13. ∗P < 0.05, ∗∗P < 0.01, ∗∗∗P < 0.001. ns, not statistically significant. Figure S2 Multiomics analysis of the relationship between CXCL13 and HRD. (A) The expression of CXCL13 protein in clinical specimens with different HRD statuses (200×). (B) Correlation between CXCL13 expression and gene expression levels of 45 HRRs. (C) Protein-protein interaction (PPI) network of related genes co-expressed with CXCL13 obtained by the STRING tool. ∗P < 0.05, ∗∗P < 0.01, ∗∗∗P < 0.001. ns, not statistically significant; HRD, homologous recombination deficiency; HRR, homologous recombination repair. Figure S3 Co-expressed genes and mutational landscape. (A–C) Correlation analysis of CXCL13 expression with gene expression of the cGAS-STING pathway. (D) The top 15 most highly mutated genes between CXCL13-H and CXCL13-L groups. Figure S4 Correlation of CXCL13 expression with tumor immune microenvironment was assessed by ESTIMATE and TIMER. (A) Significantly higher immune scores in the CXCL13-H group. (B) The association of six immune cell types with CXCL13. Figure S5 The expression of CXCL13 and its correlation with immunity in ovarian cancer cell lines. (A–D) Various algorithms were used to assess the tumor immune microenvironment (TIME) (A: xCell algorithm; B: CIBERSORT algorithm; C: QUANTISEQ algorithm; D: MCPcounter algorithm). (E) Expression of CXCL13 in the co-culture system of UWB1.289 and UWB1.289+BRCA. "U" represents UWB1.289 in the co-culture system, and "UR" represents UWB1.289+BRCA1 in the co-culture system. Figure S6 Gene expression and GSEA (apoptosis pathway) at the single-cell level. (A) Annotation of single cell fra [file mmc3.pdf]

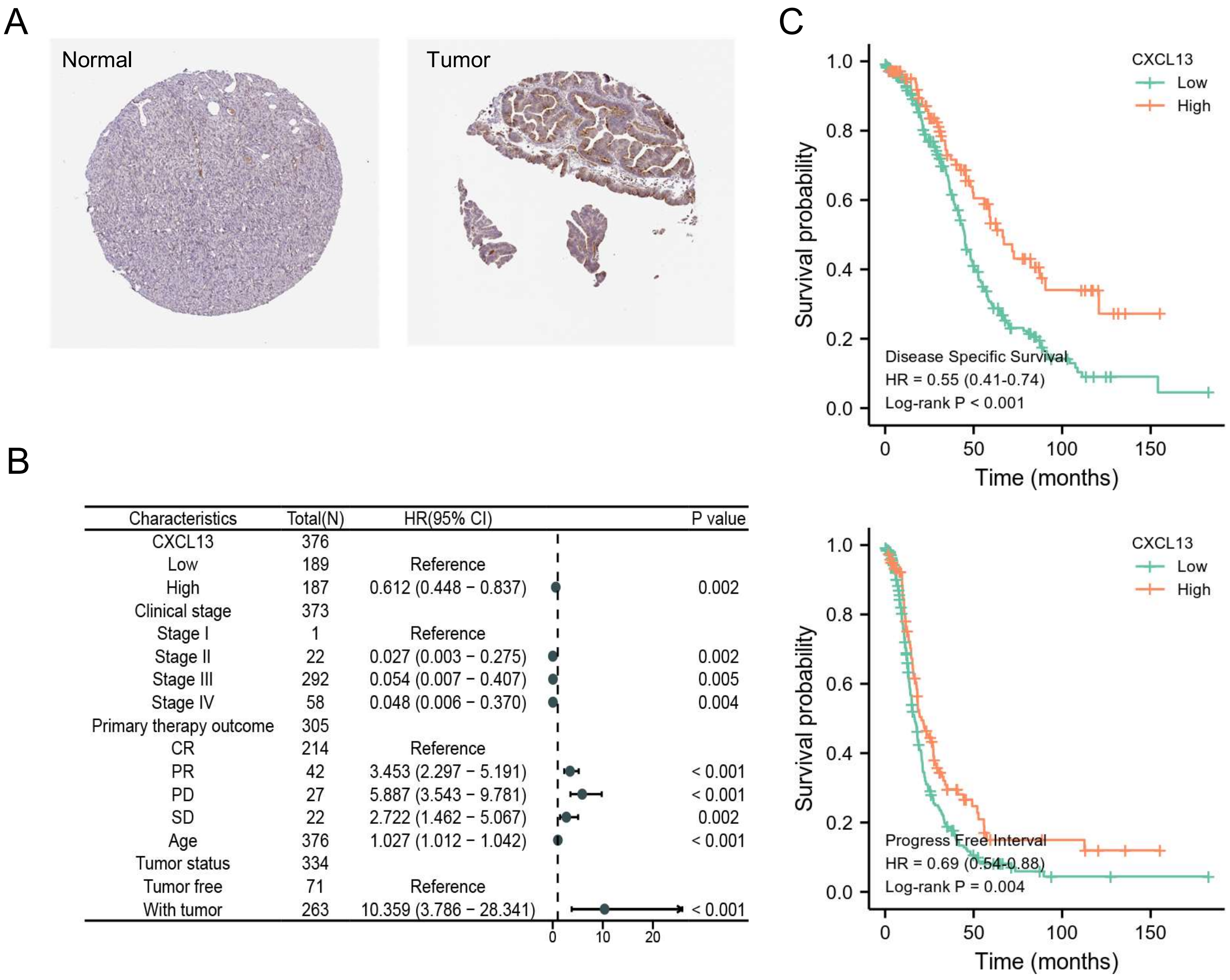
A
Normal
Tumor
B
Characteristics Total(N) HR(95% CI) P value
CXCL13 376
Low 189 Reference
High 187 0.612 (0.448 − 0.837) 0.002
Clinical stage 373
Stage I 1 Reference
Stage II 22 0.027 (0.003 − 0.275) 0.002
Stage III 292 0.054 (0.007 − 0.407) 0.005
Stage IV 58 0.048 (0.006 − 0.370) 0.004
Primary therapy outcome 305
CR 214 Reference
PR 42 3.453 (2.297 − 5.191) < 0.001
PD 27 5.887 (3.543 − 9.781) < 0.001
SD 22 2.722 (1.462 − 5.067) 0.002
Age 376 1.027 (1.012 − 1.042) < 0.001
Tumor status 334
Tumor free 71 Reference
With tumor 263 10.359 (3.786 − 28.341) < 0.001
0
10
20
C
CXCL13
Low
High
Survival probability
Disease Specific Survival
HR = 0.55 (0.41-0.74)
Log-rank P < 0.001
Time (months)
CXCL13
Low
High
Survival probability
Progress Free Interval
HR = 0.69 (0.54-0.88)
Log-rank P = 0.004
Time (months)

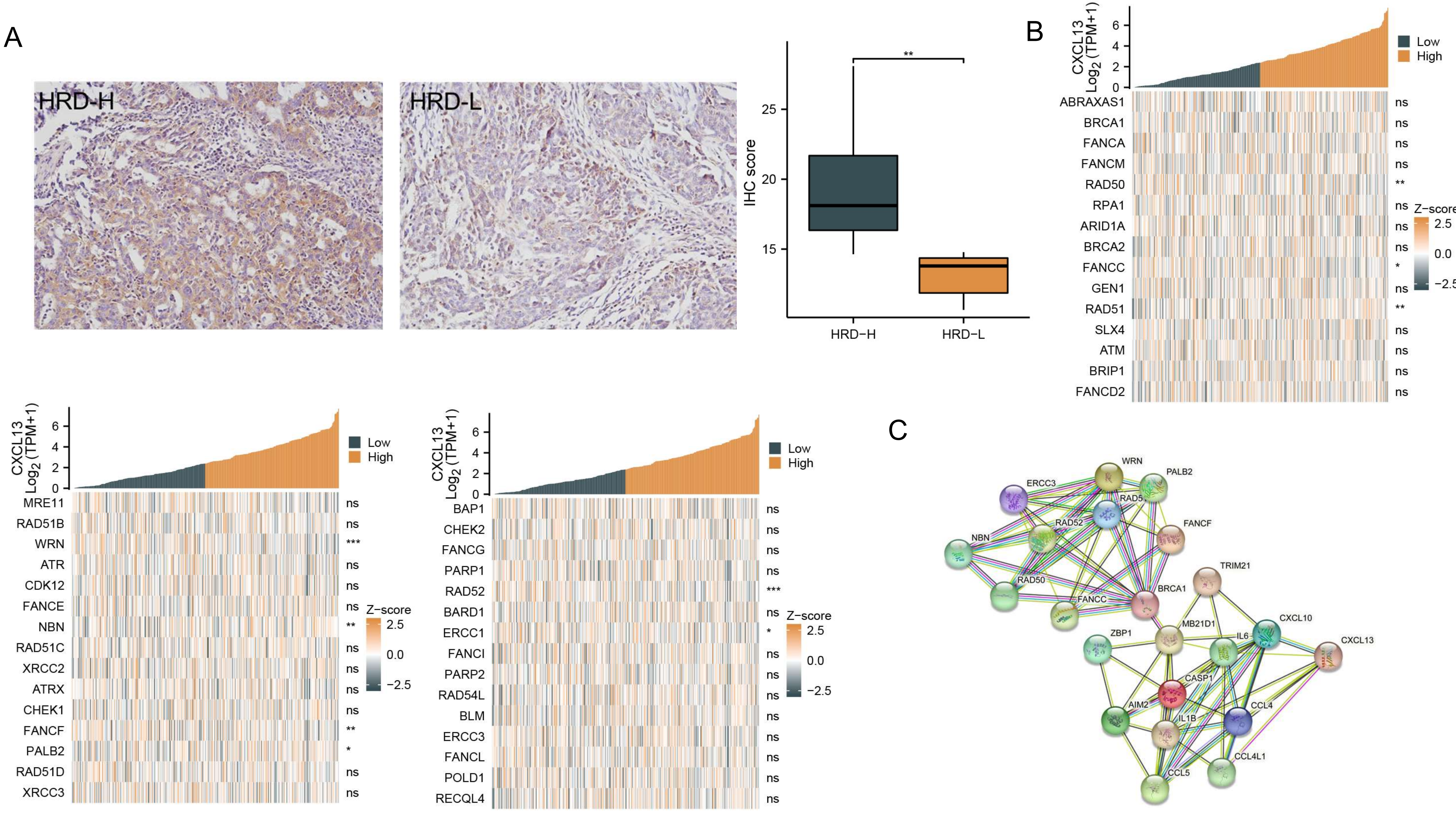
A
HRD-H
HRD-L
**
IHC score
25
20
15
HRD−H
HRD−L
B
CXCL13
Log2 (TPM+1)
6
4
2
0
Low
High
ABRAXAS1 ns
BRCA1 ns
FANCA ns
FANCM ns
RAD50 **
RPA1 ns
ARID1A ns
BRCA2 ns
FANCC *
GEN1 ns
RAD51 **
SLX4 ns
ATM ns
BRIP1 ns
FANCD2 ns
Z−score
2.5
0.0
MRE11 ns
RAD51B ns
WRN ***
ATR ns
CDK12 ns
FANCE ns
NBN **
RAD51C ns
XRCC2 ns
ATRX ns
CHEK1 ns
FANCF **
PALB2 *
RAD51D ns
XRCC3 ns
−2.5
BAP1 ns
CHEK2 ns
FANCG ns
PARP1 ns
RAD52 ***
BARD1 ns
ERCC1 *
FANCI ns
PARP2 ns
RAD54L ns
BLM ns
ERCC3 ns
FANCL ns
POLD1 ns
RECQL4 ns
C
WRN
PALB2
ERCC3
RAD51
RAD52
FANCF
NBN
TRIM21
RAD50
BRCA1
FANCC
MB21D1
CXCL10
ZBP1
IL6
CXCL13
CASP1
AIM2
CCL4
IL1B
CCL4L1
CCL5

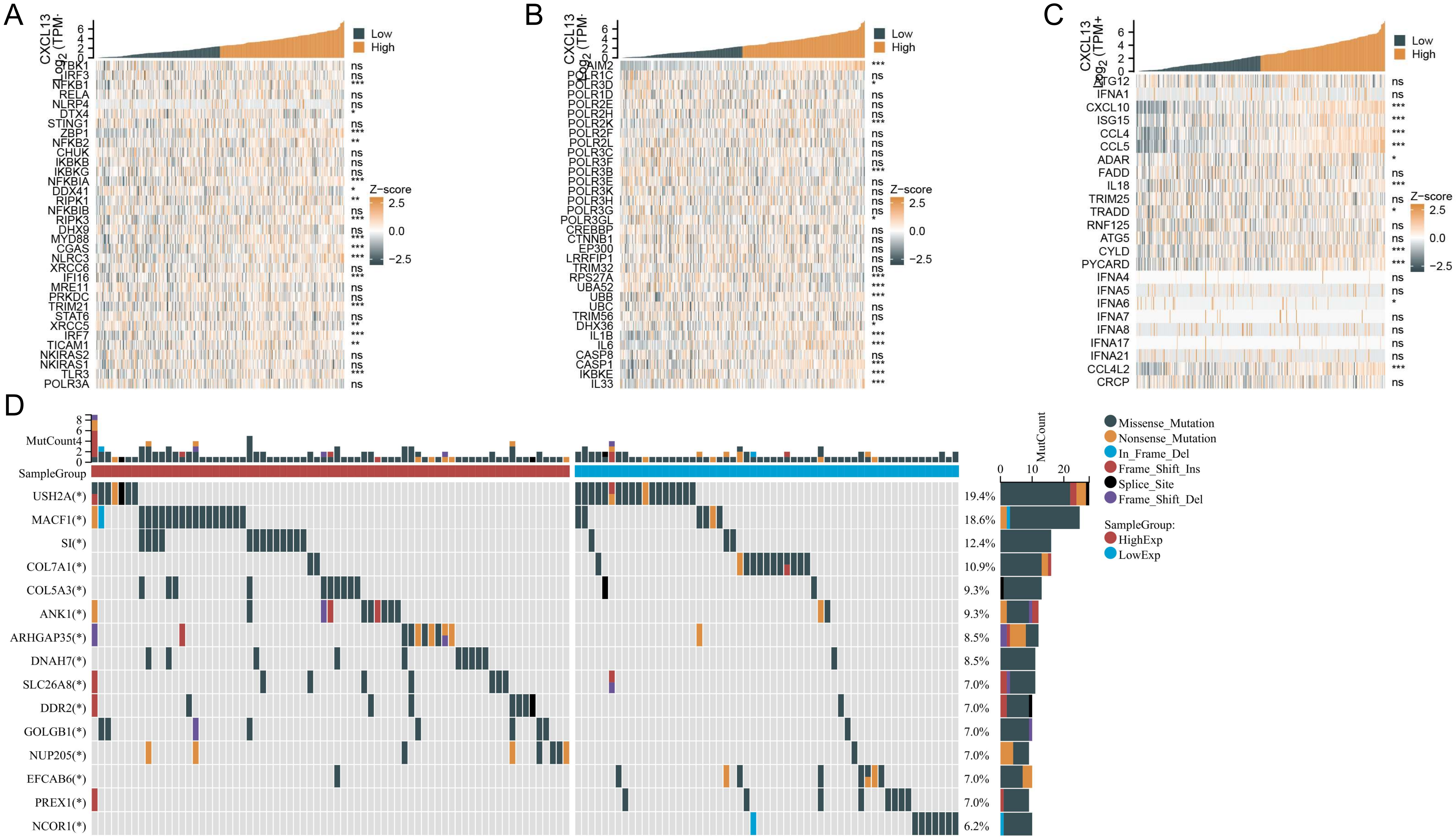
A
CXCL13 Log2 (TPM+
Low
High
TBK1 ns
IRF3 ns
NFKB1 ***
RELA ns
NLRP4 ns
DTX4 *
STING1 ns
ZBP1 ***
NFKB2 **
CHUK ns
IKBKB ns
IKBKG ns
NFKBIA ***
DDX41 *
RIPK1 **
NFKBIB ns
RIPK3 ***
DHX9 ns
MYD88 ***
CGAS ***
NLRC3 ***
XRCC6 ns
IFI16 ***
MRE11 ns
PRKDC ns
TRIM21 ***
STAT6 ns
XRCC5 **
IRF7 ***
TICAM1 **
NKIRAS2 ns
NKIRAS1 ns
TLR3 ***
POLR3A ns
Z-score
2.5
0.0
−2.5
B
AIM2 ***
POLR1C ns
POLR3D *
POLR1D ns
POLR2E ns
POLR2H ns
POLR2K ***
POLR2F ns
POLR2L ns
POLR3C ns
POLR3F ns
POLR3B ***
POLR3E ns
POLR3K ns
POLR3H ns
POLR3G ns
POLR3GL *
CREBBP ns
CTNNB1 ns
EP300 ns
LRRFIP1 ns
TRIM32 ns
RPS27A ***
UBA52 ***
UBB ***
UBC ns
TRIM56 ns
DHX36 *
IL1B ***
IL6 ***
CASP8 ns
CASP1 ***
IKBKE ***
IL33 ***
C
ATG12 ns
IFNA1 ns
CXCL10 ***
ISG15 ***
CCL4 ***
CCL5 ***
ADAR *
FADD ns
IL18 ***
TRIM25 ns
TRADD *
RNF125 ns
ATG5 ns
CYLD ***
PYCARD ***
IFNA4 ns
IFNA5 ns
IFNA6 *
IFNA7 ns
IFNA8 ns
IFNA17 ns
IFNA21 ns
CCL4L2 ***
CRCP ns
D
MutCount
SampleGroup
USH2A(*) 19.4%
MACF1(*) 18.6%
SI(*) 12.4%
COL7A1(*) 10.9%
COL5A3(*) 9.3%
ANK1(*) 9.3%
ARHGAP35(*) 8.5%
DNAH7(*) 8.5%
SLC26A8(*) 7.0%
DDR2(*) 7.0%
GOLGB1(*) 7.0%
NUP205(*) 7.0%
EFCAB6(*) 7.0%
PREX1(*) 7.0%
NCOR1(*) 6.2%
Missense_Mutation
Nonsense_Mutation
In_Frame_Del
Frame_Shift_Ins
Splice_Site
Frame_Shift_Del
SampleGroup:
HighExp
LowExp

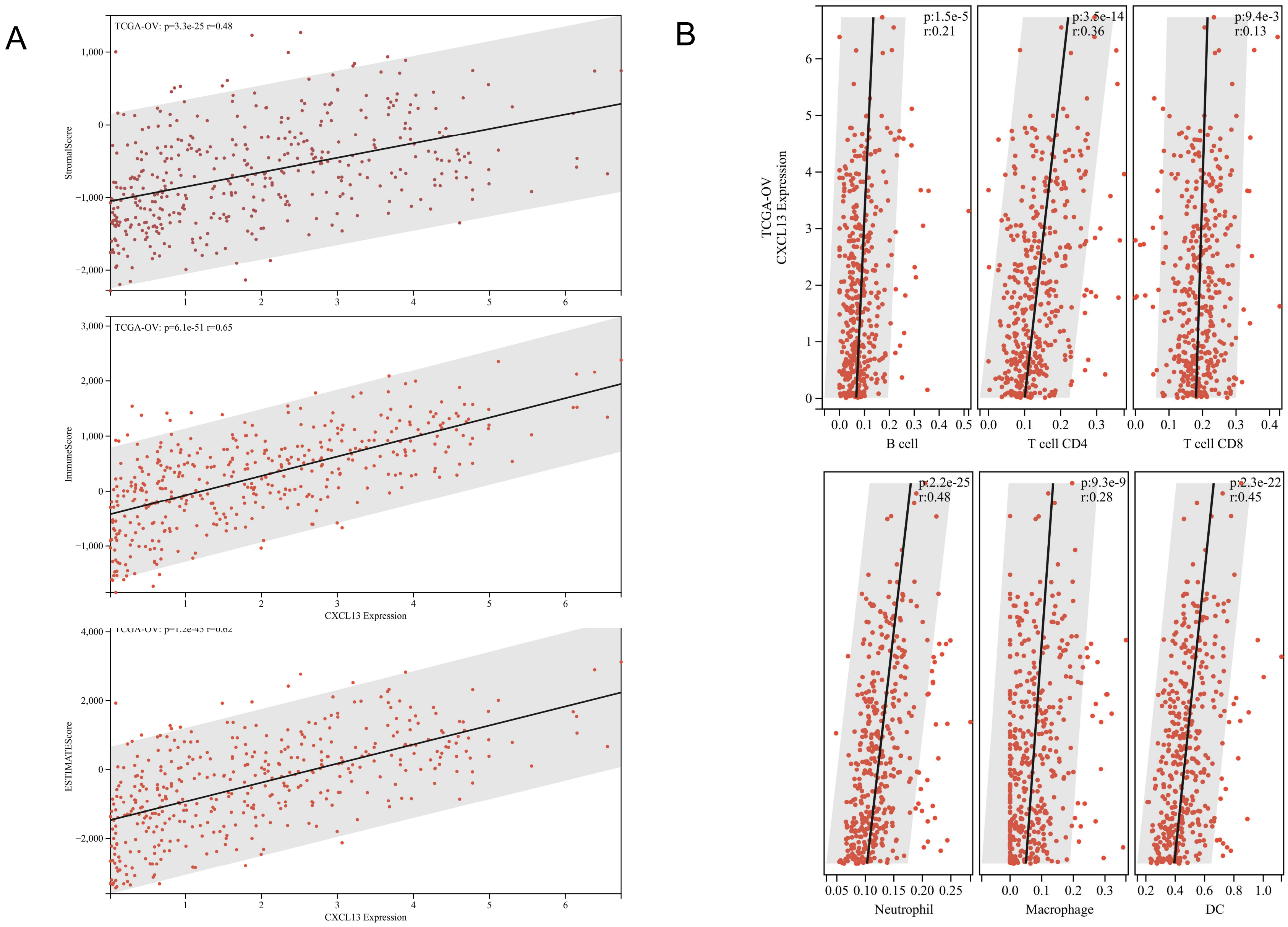
A
TCGA-OV: p=3.3e-25 r=0.48
StromalScore
TCGA-OV: p=6.1e-51 r=0.65
ImmuneScore
CXCL13 Expression
ESTIMATEScore
CXCL13 Expression
B
TCGA-OV
CXCL13 Expression
p:1.5e-5
r:0.21
B cell
p:3.5e-14
r:0.36
T cell CD4
p:9.4e-3
r:0.13
T cell CD8
p:2.2e-25
r:0.48
Neutrophil
p:9.3e-9
r:0.28
Macrophage
p:2.3e-22
r:0.45
DC

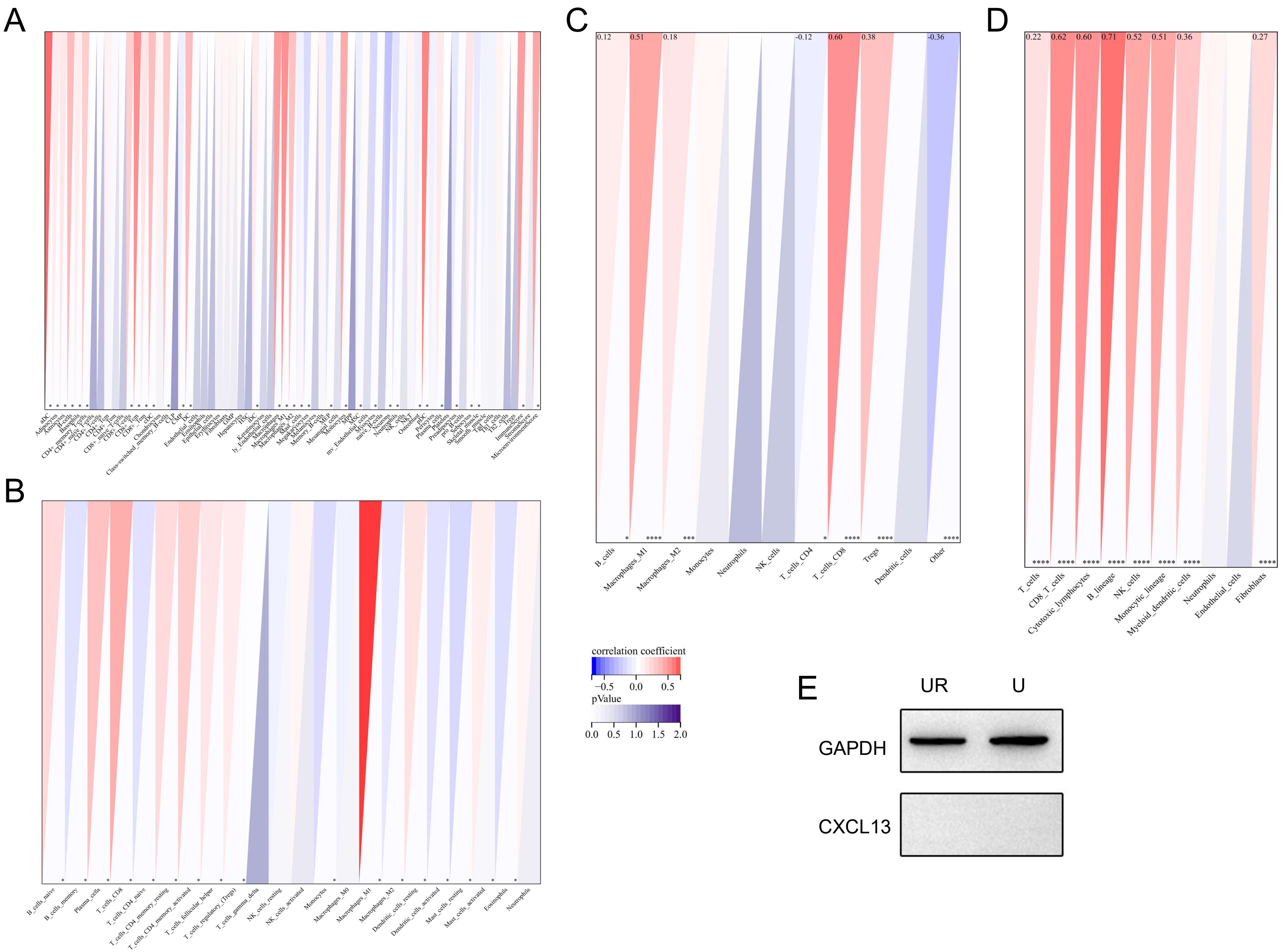
A
B
C
0.12
0.51
0.18
-0.12
0.60
0.38
-0.36
B_cells
Macrophages_M1
Macrophages_M2
Monocytes
Neutrophils
NK_cells
T_cells_CD4
T_cells_CD8
Tregs
Dendritic_cells
Other
D
0.22
0.62
0.60
0.71
0.52
0.51
0.36
0.27
T_cells
CD8_T_cells
Cytotoxic_lymphocytes
B_lineage
NK_cells
Monocytic_lineage
Myeloid_dendritic_cells
Neutrophils
Endothelial_cells
Fibroblasts
correlation coefficient
−0.5 0.0 0.5
pValue
0.0 0.5 1.0 1.5 2.0
E
UR
U
GAPDH
CXCL13

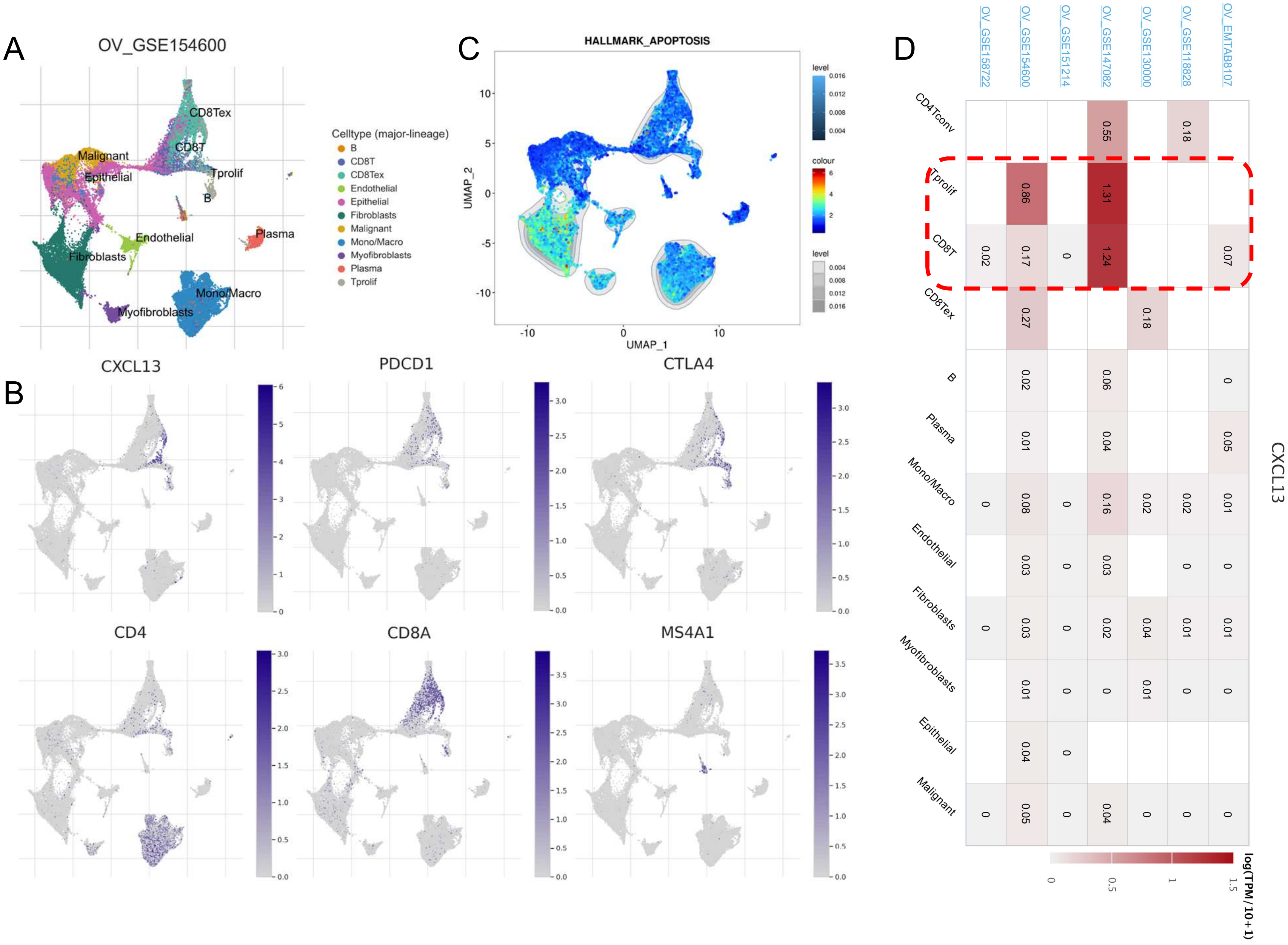
A
OV_GSE154600
CD8Tex
CD8T
Malignant
Epithelial
Tprolif
B
Endothelial
Plasma
Fibroblasts
Mono/Macro
Myofibroblasts
Celltype (major-lineage)
B
CD8T
CD8Tex
Endothelial
Epithelial
Fibroblasts
Malignant
Mono/Macro
Myofibroblasts
Plasma
Tprolif
B
CXCL13
PDCD1
CTLA4
CD4
CD8A
MS4A1
C
HALLMARK_APOPTOSIS
UMAP_2
UMAP_1
level
colour
D
OV_GSE158722
OV_GSE154600
OV_GSE151214
OV_GSE147082
OV_GSE130000
OV_GSE118828
OV_EMTAB8107
CD4Tconv
Tprolif
CD8T
CD8Tex
B
Plasma
Mono/Macro
Endothelial
Fibroblasts
Myofibroblasts
Epithelial
Malignant
CXCL13
log(TPM/10+1)

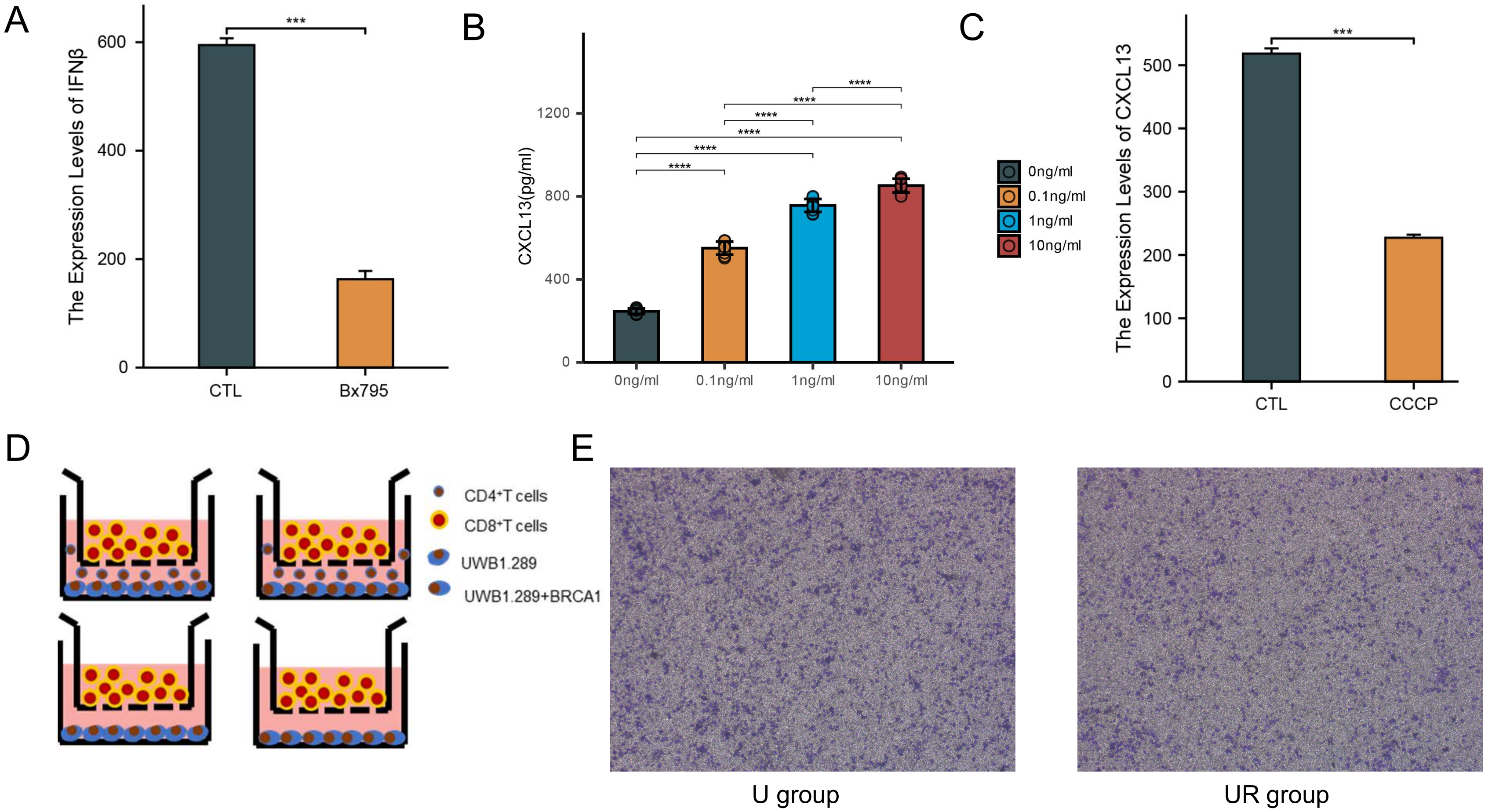
A
The Expression Levels of IFNβ
0
200
400
600
***
CTL
Bx795
B
CXCL13(pg/ml)
0
400
800
1200
****
0ng/ml
0.1ng/ml
1ng/ml
10ng/ml
C
The Expression Levels of CXCL13
0
100
200
300
400
500
***
CTL
CCCP
D
CD4+T cells
CD8+T cells
UWB1.289
UWB1.289+BRCA1
E
U group
UR group
